# Forecasting and Surveillance of COVID-19 Spread Using Google Trends: Literature Review

**DOI:** 10.3390/ijerph191912394

**Published:** 2022-09-29

**Authors:** Tobias Saegner, Donatas Austys

**Affiliations:** Department of Public Health, Institute of Health Sciences, Faculty of Medicine, Vilnius University, M. K. Čiurlionio 21/27, LT-03101 Vilnius, Lithuania

**Keywords:** COVID-19, forecasting, surveillance, Google Trends

## Abstract

The probability of future Coronavirus Disease (COVID)-19 waves remains high, thus COVID-19 surveillance and forecasting remains important. Online search engines harvest vast amounts of data from the general population in real time and make these data publicly accessible via such tools as Google Trends (GT). Therefore, the aim of this study was to review the literature about possible use of GT for COVID-19 surveillance and prediction of its outbreaks. We collected and reviewed articles about the possible use of GT for COVID-19 surveillance published in the first 2 years of the pandemic. We resulted in 54 publications that were used in this review. The majority of the studies (83.3%) included in this review showed positive results of the possible use of GT for forecasting COVID-19 outbreaks. Most of the studies were performed in English-speaking countries (61.1%). The most frequently used keyword was “coronavirus” (53.7%), followed by “COVID-19” (31.5%) and “COVID” (20.4%). Many authors have made analyses in multiple countries (46.3%) and obtained the same results for the majority of them, thus showing the robustness of the chosen methods. Various methods including long short-term memory (3.7%), random forest regression (3.7%), Adaboost algorithm (1.9%), autoregressive integrated moving average, neural network autoregression (1.9%), and vector error correction modeling (1.9%) were used for the analysis. It was seen that most of the publications with positive results (72.2%) were using data from the first wave of the COVID-19 pandemic. Later, the search volumes reduced even though the incidence peaked. In most countries, the use of GT data showed to be beneficial for forecasting and surveillance of COVID-19 spread.

## 1. Introduction

Coronavirus Disease (COVID-19), caused by the novel acute respiratory syndrome coronavirus 2 (SARS-CoV-2), is an infectious disease with high virulence and a high proportion of asymptomatic cases, which, together with other factors such as a long period from infection to the onset of the symptoms, symptoms’ similarity to a regular cold, and continuous social interactions, led to a worldwide virus outbreak [1,2,3].

Early detection of COVID-19 outbreaks is crucial for multiple reasons: (i) to prepare hospitals and staff, including efficiently allocating protective gear and medical equipment [4], as well as testing tents and setting up IT infrastructure (setting up electronic health information systems for patient registration and databases); (ii) to prepare governments for actions, such as imposing curfew, ordering equipment, and drawing up guidelines for businesses and events; (iii) to improve public messaging and warn people about the risks and their prevention; (iv) to prevent further spread of infection [4] by imposing local quarantine or taking other preventive measures. The probability of future COVID-19 waves remains high [5]; thus, COVID-19 surveillance and forecasting remain important.

Online search engines harvest vast amounts of data from the general population in real time. Importantly, many of them, including the most popular Google search engine, make these data publicly accessible. This raises the interest of using such data for surveillance and forecasting of disease outbreaks [6]. Among internet-based tools for analysis of search queries used to search for specific information, the most acclaimed one is Google Trends (GT) [7,8]. As stated by other researchers, GT can be employed to solve public health issues as it provides valuable information about current concerns and health-related problems in general society, especially in the field of infectious diseases [7] and, therefore, could be used for prediction of upcoming disease waves.

GT as a prediction tool that has been used for many different diseases in the past two decades, including Influenza [9], Zika virus disease [10], Middle East Respiratory Syndrome (MERS) [11], and Malaria [12]. These studies provided diverging results, which makes it difficult to make generalized conclusions about a possibility to use GT for prediction and surveillance of infectious diseases. When it comes to COVID-19, it is important to assess GT’s ability to detect changes in numbers of people who possibly do not perform COVID-19 tests, but nonetheless feel symptoms or who suspect that they had contact with an infected person and can infect others. This could be used for prediction of COVID-19 outbreaks. Therefore, **the aim** of this study was to review the literature about the possible use of GT for COVID-19 surveillance and prediction of its outbreaks.

## 2. Materials and Methods

This literature review included articles published within 2 years from the beginning of the pandemic until February 2022. The PubMed search engine was used to search for scientific publications.

### Inclusion and Exclusion Criteria

The search phrases used for the search query were “Google Trends” AND “COVID-19”. For the initial search of publications, we did not use any time, language, publication type, or other criteria filters. The initial search yielded 301 results. All publications were reviewed for the following inclusion criteria: (i) primary original articles addressing the usage of GT for COVID-19 prediction and/or surveillance; (ii) articles available in full text for our institutional network; exclusion criteria: (i) publications that had only part of the search phrase in the title or the snippet of the abstract which made us suppose that the publication was not about usage of GT tool; (ii) publications with type review, letter, comment, correspondence, or presentation; (iii) publications written in any other language than English; (iv) publications where data obtained from other sources than GT (e.g., WikiTrends, Twitter, etc.) were analyzed.

**Figure 1:**
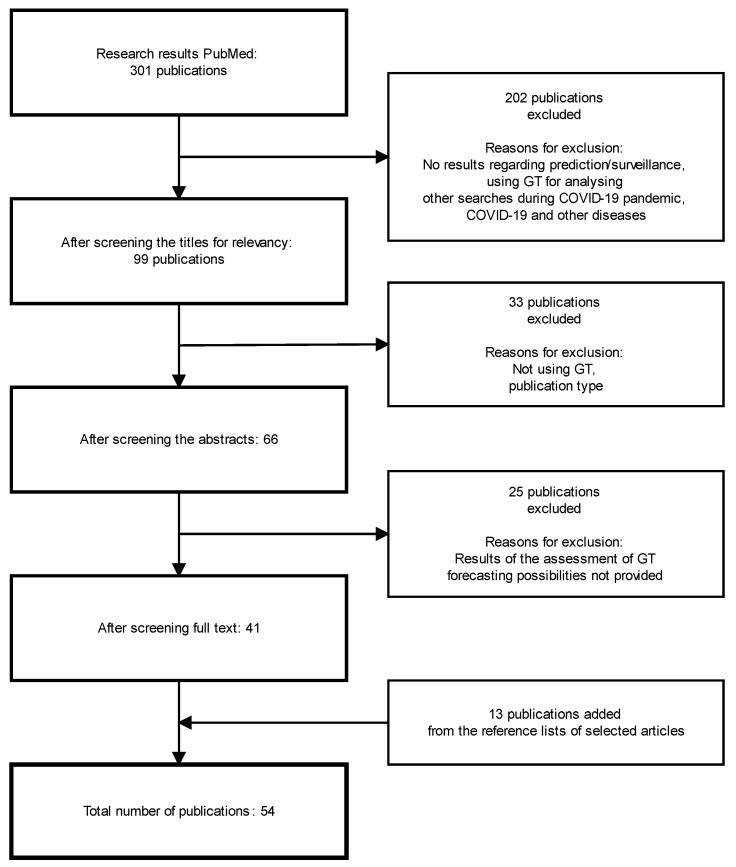
Selection process of the articles to review.

Firstly, with respect to inclusion and exclusion criteria, the publication titles were screened to determine if the publication could possibly fit the scope of this review, which ruled out 202 articles found during the initial search. As a second step, 99 article abstracts of the selected publications were screened to verify the relevance of the publication, ruling out 33 publications. The full text was downloaded only if an abstract showed that the publication might be relevant to this review. Full texts of 66 articles were then analyzed to include only those articles which provided the results of the assessment of GT forecasting possibilities for COVID-19 disease. In addition, the reference lists of included publications were reviewed according to the same criteria for those not uncovered with the initial search. After completing all these steps and removing duplicates, we concluded with 44 articles meeting all the criteria (Figure 1).

From each included publication, we extracted such data as year of publication, short description of the main findings, country where GT data were collected, keywords used by people in that country, period of data collection for GT analysis, and the statistical analysis method(s) used to analyze the data.

## 3. Results

Most of the studies (83.3%) included in this review showed positive results of the possible use of GT for forecasting COVID-19 outbreaks (Table 1 and Table 2). Most of the publications with positive results were performed in Western countries—mostly in Europe [8,13,14,15,16,17,18,19,20,21,22,23,24,25,26,27,28,29,30,31,32,33,34,35,36,37,38,39,40,41] (55.5%), less in the USA [5,7,8,13,14,17,20,22,23,25,27,30,31,34,36,37,38,42,43,44,45,46,47,48,49,50,51,52] (51.9%), Australia [8,14,22,23,25,30,34,38] (14.8%), and Canada [22,25,30,34,38,48] (11.1%). The rest (50%) of the studies were performed in the Middle East [8,13,18,22,23,25,37,53,54,55] (18.5%), India [20,22,23,25,30,37,56,57] (14.8%), and China [8,22,25,35,37,58,59] (13%). A total of 46.3% of the included studies analyzed data from multiple countries and 53.7% analyzed GT data in single countries. Most of the studies analyzed GT data in the USA [5,7,42,43,44,45,46,47,49,50,51,52] (22.2%), followed by Italy [15,21,28,29,40] (9.3%), India [56,57] (3.7%), Iran [53,55] (3.7%), Germany [19,33] (3.7%), China [58,59] (3.7%), Spain [39] (1.9%), and Taiwan [60] (1.9%).

### 3.1. Differences between Countries

Most of GT COVID-19 related analyses were performed in English-speaking countries: mostly the USA [5,7,8,13,14,17,20,22,23,25,27,30,31,34,36,37,38,42,43,44,45,46,47,48,49,50,51,52] (51.9%), as well as the United Kingdom [8,13,14,17,18,20,22,23,24,25,26,30,31,32,34,37,38,61] (33.3%), Australia [8,14,22,23,25,30,34,38] (14.8%), and Canada [22,25,30,34,38,48] (11.1%). Similarly, more studies were performed in bigger countries, i.e., those with many residents, as opposed to smaller ones. Moreover, it is seen that GT data analysis was performed more in high-income countries compared to medium- and low-income ones.

### 3.2. Time Periods

GT seemed to have a higher prediction capability during the first wave of the COVID-19 pandemic (most of the studies (72.2%) took GT data from 01 2020 to 05 2020). The majority of studies reviewed in this article used GT data obtained in 2020 (some starting December 2019) with only four extending their GT data collection to previous years [23,30,52,64] for comparison.

### 3.3. Keywords

The most frequently used keyword was “coronavirus” (53.7%), followed by “COVID-19” (31.5%) and “COVID” (20.4%). Other variations included “corona”, “SARS-CoV2”, and “COVID19” (or other variations). Specific symptoms showed to be less frequently used in Google searches; however, searches relating to loss of smell and taste (31.5%) were rather common as well.

### 3.4. More Complex Analysis Methods of GT Data

There were some publications with more complex methods used for statistical analysis of GT data (Table 2). Long short-term memory [20,53] (3.7%), random forest regression [22,25] (3.7%), Adaboost algorithm [21] (1.9%), autoregressive integrated moving average (ARIMA), error, trend and seasonality (ERS), neural network autoregression (NNA) [23] (1.9%), and vector error correction modeling [50] (1.9%) were described as methods of analysis. The findings of those studies showed that GT significantly improved the predictive capability of the methods used in the analysis and could be used in the future with even higher predictability as more data become available [25,53].

### 3.5. Negative Results of GT Use for COVID-19 Prediction and Surveillance

Nine publications (Table 3) showed negative results of GT use in COVID-19 surveillance and/or prediction. Most of them [26,28,31,57,64] stated that the correlations between GT search queries and COVID-19 cases in those countries were present because of media coverage [31,57,64] or announcements by governments and/or WHO [26,28]. A high variation in correlations between COVID-19 incidence and internet searches was identified as well [27,29], showing that GT data are not a reliable source for COVID-19 prediction and surveillance.

## 4. Discussion

### 4.1. Differences between Countries

One possible reason why there were more studies performed in high-income countries compared to low-income ones could be the lack of IT infrastructure—only 50% of individuals in low- and middle-income countries are using internet [65] as opposed to almost 90% in high-income countries [66], thus allowing people to search for information easily. For example, even though India is the second country in the world in internet user numbers, only 36% percent of its population use internet monthly [67] as opposed to over 90% in the USA [68,69] or 92% of households in Europe [70].

### 4.2. Time Periods

It was seen that most of the publications with positive results were using data from the first wave of the COVID-19 pandemic. Later, the search volumes reduced [33] even though the incidence peaked. This could be explained by people’s initial fear and lack of knowledge about the disease—symptoms, as well as protection measures, were more searched during the first wave. Later, such information became more widely known—not only people learned while searching themselves, but there were plenty of announcements from the governments as well as WHO. Naturally, people lost interest in following such news [71] in addition to getting “tired” of lockdowns.

The strong public interest decline in COVID-19-related issues might cause a big public health challenge to distribute relevant information regarding the newest developments in disease treatment and prevention measures throughout the whole pandemic [33].

### 4.3. Risk Communication

Four publications [33,38,72,73] identified during the PubMed database search were not about prediction or surveillance of COVID-19 using GT data, rather about public interest in the pandemic and risk communication during the outbreaks. Those studies have shown increased amount of search queries after first case announcement [33,73] and such events such as local COVID-19 transmission, approval and implementation of testing, social-distancing campaign, face mask shortage, and announcements by WHO [72].

As people’s interest peaked, it would be sensible to spread scientific information and promote preventative measures, as well as prevent misinformation in this exact time period. It would be beneficial to target social media, where misinformation spreads the fastest and people feel properly informed while reading non-expert opinions and statements. In addition, the decline in interest should be met with informational campaigns to ensure proper information spreads [38] as well as showing people where to search for information and how to distinguish facts discovered by scientists from non-expert opinions.

### 4.4. Language

Our study reviews publications made in many different countries, which results in different search terms. Several studies [38] indicated the importance of ‘related query analysis’ prior to further analysis since it can point out the most relevant search terms.

Furthermore, there were many multi-country studies where the search terms were translated, thus potentially resulting in lost nuances of the meaning as well as some overlay [13].

### 4.5. Complex Analysis Methods of GT Data

Several studies (Table 2) incorporated GT data in their machine learning algorithms. Results of these studies show that such method was able to successfully predict an increase in COVID-19 cases in a large number of countries 7 days in advance [22,25]. Furthermore, data of previous incidence of COVID-19 and GT were combined, which showed improved performance of the prediction models compared to previous ones which used incidence data alone [23].

When conventional metrics (numbers of cases and deaths) were combined with interest-over-time values, the prediction ability of the models increased further [23]. Rabiolo et al. have identified two principal components, which allowed to reduce data dimensionality and summarize the information into two components, thus providing a flexible approach which allows the variables of interest to change and use the same models to investigate different research questions in the future [23]. Moreover, an additional advantage is that the performance of these models can be further improved as more data become available over time and can reflect the current situation [25,53]. In addition, the models could have other uses than predicting COVID-19 numbers, e.g., assessing people’s awareness and engagement, thus allowing health authorities to use these data for measuring the effectiveness of the information spread [53], which is crucial especially when information fatigue is present [69,72].

### 4.6. Negative Findings

Few studies showed that GT data could not be used for COVID-19 prediction and/or surveillance. According to the authors of those studies, WHO and/or local government announcements had a major influence on search trends [26,57] and that GT is more efficient in tracking a new disease outbreak when media coverage of that disease is absent [26]. However, this was not possible to test during this pandemic since WHO, as well as governments and officials, started communication regarding the novel coronavirus even before WHO announced it as a pandemic. Furthermore, the authors suggest that online searches simply overlap with the increase in COVID-19 cases and related deaths since big media announcements are made at the same time as increase in incidence happens [26] or were a result of information-seeking curiosity [57].

### 4.7. Strengths

Many studies have made analyses in multiple countries and obtained the same results for the majority of them [8,13,37,38,61,62], thus showing the robustness of the chosen methods. Furthermore, Google search data are easy to obtain, more dynamic, and available compared to traditional data sources, such as data from governmental institutions, health authorities, etc., as well as represent current moods of the population and can be obtained during multiple periods [53]. As the relevant search terms can change over time, it is possible to investigate GT data repeatedly and incorporate the new terms and newly available data into the prediction models, thus improving the outcome. Even more improvement in prediction can be reached when search terms with higher correlation values are used for the analysis [20].

### 4.8. Limitations of the Possible Use of GT

One of the main limitations noted by the authors of the studies analyzed was the short timeframe taken for the analysis [25,38]. The positive results obtained from the first COVID-19 wave could have been due to the virus being new and interesting to the society, including mass media. Possibly, these factors resulted in an increase in searches using Google and other search engines. Furthermore, such methods must account for misspellings and possible other search terms [38] as well the fact that Google might not be the main search engine for different groups of people [23,25,26,38]. One more disadvantage lies in the data (incidence and death rate) which are used to compare it with the ones obtained from Google. Different countries have different testing policies, as well as death reports, thus making it impossible to have a standardized number [26,38]. Moreover, COVID-19 reports in other countries and media coverage everywhere around the world, as well as people’s curiosity, might have influenced the increase in searches [13,25]. It was not possible to take into account many of the social and demographic factors (gender, age, education level, literacy) of the searchers [13,26]. One could speculate that older people are not represented in the search volumes, even though they are one of the mostly vulnerable groups for COVID-19. They, together with children, as well as people living in areas with poor internet connection, cannot be studied with this strategy, i.e., using GT data to make predictions, thus making it implausible for countries with large rural areas [23]. Similarly, the symptom similarity and prevention methods between COVID-19 and influenza might not allow to differentiate between the two [25,57], potentially showing higher search volumes and influencing the predictions.

### 4.9. Limitations of the Review

The limitations of this review include potentially missing results published in relevant publications written in any language other than English. Furthermore, focusing only on Google Trends can possibly exclude other internet-based tools useful for COVID-19 prediction and surveillance. Similarly, we included only those articles that were accessible to our institutional network which could exclude some relevant studies from this review. In addition, despite the fact that we used the name of the tool analyzed in this review (Google Trends) and the name of the disease (COVID-19) as keywords for the search of the publications, we could have missed some publications. Possibly, adding more keywords to the search query could help find more publications and this should be addressed in future reviews.

## 5. Conclusions

The majority of the studies analyzed in this paper have reported positive findings regarding prediction and surveillance of COVID-19 cases using data obtained from Google Trends. Incorporating GT data into various COVID-19 forecasting algorithms could increase their prediction capabilities. Further analyses using data obtained during later time periods are needed to further evaluate the forecasting capabilities of GT when the mass media calms down.

## Figures and Tables

**Table 1:** Publications with positive results of GT use for COVID-19 prediction and surveillance.

Author and Year	The Main Findings about Google Trends	Country	Period	Keywords
Husnayain, Fuad, Su (2020) [60]	GT can be used for public restlessness monitoring towards COVID-19 pandemic 1–3 days before the increase in confirmed cases.	TW	12 2019–02 2020	Coronavirus, hand wash, face masks
Walker, Hopkins, Surda (2020) [13]	Strong correlation between smell-related information search frequency and onset of COVID-19 infection.	IT, ES, UK, US, DE, FR, NL, IR	12 2019–03 2020	Smell, loss of smell, anosmia, hyposmia, olfaction, taste, loss of taste, dysgeusia. The keywords were automatically translated to national languages of study countries.
Mavragani (2020) [24]	Significant correlations between online interest of coronavirus and COVID-19 cases and deaths.	IT, ES, FR, DE, UK	01 2020–03 2020	Coronavirus
Venkatesh and Gandhi (2020) [56]	Google Web, together with other internet-based tools might be useful in predicting COVID-19 outbreaks 2–3 weeks earlier than conventional disease surveillance.	IN	01 2020–04 2020	Coronavirus, COVID, COVID-19, corona, virus
Kurian, Bhatti, Alvi, Ting, Storlie, Wilson, Shah, Liu, Bydon (2020) [7]	The information obtained from GT precedes COVID-19 outbreaks. This information could allow better preparation and planning of health care systems.	US	01 2020–04 2020	COVID symptoms, coronavirus symptoms, sore throat + shortness of breath + fatigue + cough, coronavirus testing center, loss of smell, Lysol (sanitizer), antibody, face mask, coronavirus vaccine, COVID stimulus check
Panuganti, Jafari, MacDonald, DeConde (2020) [42]	Google search of fever and shortness of breath are better indicators of COVID-19 incidence than anosmia.	US	01 2020–04 2020 (excluding a short timeframe (March 22 to March 24))	COVID, coronavirus, COVID-19, SARS-CoV-2, and COVID19, nonsmell symptoms of COVID-19 (shortness of breath, fatigue, cough, and fever) loss of smell, anosmia, lose smell, sense of smell, cannot smell, can’t smell and hyposmia, nasal irrigation and sinus rinse, (dysgeusia, taste change and taste loss, COVID, coronavirus, COVID-19, SARS-CoV-2, and COVID19), (shortness of breath, fatigue, cough, and fever), and smell loss anosmia, loss of smell, reduced smell, decreased smell, lose your sense of smell, lost sense of smell, decreased sense of smell, decrease your sense of smell, decreased my sense of smell, reduce your sense of smell, reduced my sense of smell, reduced sense of smell, loss of sense of smell, loss of smell, hyposmia
Mavragani and Gkillas (2020) [43]	Significant correlation found between GT search queries and COVID-19 incidence.	US	03 2020–04 2020	coronavirus (virus) and coronavirus (search term)
Higgins, Wu, Sharma, Illing, Rubel, Ting, Alliance (2020) [35]	Many search terms showed significant correlations with COVID-19 cases and mortality rate.	CN, US, IT, ES	01 2020–04 2020	Real world deaths, Coronavirus, COVID-19, Fever, SOB, Cough, Sputum, Anosmia, Dys/ageusia, Nasal congestion, Rhinorrhea, Sneezing, Sore throat, Headache, Myalgia, Chest pain, Eye pain, Diarrhea
Ahmad, Flanagan, Staller (2020) [44]	Google searches for gastrointestinal symptoms preceded the increase in COVID-19 cases in a predictable manner.	US	01 2020–04 2020	ageusia, abdominal pain, loss of appetite, anorexia, diarrhea, and vomiting
Cherry, Rocke, Chu, Liu, Lechner, Lund, Kumar (2020) [36]	GT data containing searches related to loss of smell could potentially identify COVID-19 outbreaks.	IT, ES, FR, BR, US	02 2020–05 2020	loss of sense of smell, loss of sense of taste, sense of smell, sense of taste
Cousins, Cousins, Harris, Pasquale (2020) [5]	Identifiable patterns in internet searches could predict COVID-19 outbreaks, although stochastic changes in search intensity can alter these predictions.	US	01 2020–04 2020	463 unique search queries. Appendix A.
Sharma and Sharma (2020) [37]	A positive correlation between COVID-19 cases and GT values has been recorded.	US, ES, IT, FR, UK, CN, IR, IN	03 2020–04 2020	COVID-19
Schnoell, Besser, Jank, Bartosik, Parzefall, Riss, Mueller, Liu (2021) [38]	Clear correlation found between GT data and COVID-19 incidence. GT data might be useful in selecting the best timing for web-based COVID-19-specific information and prevention measures.	AU, BR, CA, DE, IT, ZA, ROK, ES, UK, US	01 2020–06 2020	Coronavirus, corona
Jimenez, Estevez-Rebored, Santed, Ramos (2020) [39]	Significant correlation found between the rise of COVID-19 incidences and GT search queries with a lag of 11 days.	ES	02 2020–05 2020	cansancio, which translates as fatigue; coronavirus, COVID 19, covid 19, and COVID19; diarrea, which translates as diarrhea; dolor de garganta, which translates as sore throat; fiebre, which translates as fever; neumonia, which translates as pneumonia and was searched without an accent due to being more relevant; perdida de olfato, which translates as lost sense of smell and was also searched without an accent; tos, which translates as cough
Lippi, Mattiuzzi, Cervellin (2020) [40]	Significant correlations found between GT search data and newly diagnosed COVID-19 cases with a 3-week lag.	IT	02 2020–05 2020	tosse (i.e., cough), febbre (i.e., fever), and dispnea (i.e., dyspnea)
Strzelecki, Azevedo, Albuquerque (2020) [41]	There was a correlation between COVID-19 spread and GT search data for personal protective gear and hand hygiene.	PL, PT	01 2020–06 2020	máscara cirúrgica (face mask), desinfetante (sanitizer), and álcool (alcohol)
Badell-Grau, Cuff, Kelly, Waller-Evans, Lloyd-Evans (2020) [14]	Strong correlations found between COVID-19-related search terms and cases and mortality rates from COVID-19.	AU, DE, IT, ES, UK, US	11 2019–04 2020	keywords used in three categories and four languages: Government Policy, Medical Interventions, and Misinformation
Rajan, Sharaf, Brown, Sharaiha, Lebwohl, Mahadev (2020) [45]	GT data could be used to identify active disease transmission areas in the beginning of new outbreaks.	US	10 2019–05 2020	diarrhea, nausea, vomiting, and abdominal pain. The terms fever and cough were included as positive controls. The term constipation was included as a negative control.
Xie, Tan, Li (2020) [58]	Monitoring internet search activity could prevent and control the epidemic and rumors around it.	CN	01 2020–02 2020	Coronavirus
Hartwell, Greiner, Kilburn, Ottwell (2020) [46]	GT data relating to the public interest of COVID-19 preventative measures correlated with stay-at-home expiration dates and decreased new COVID-19 cases after that expiration. In addition, states with higher interest in preventative measures had higher COVID-19-related deaths per capita and higher case-fatality rates.	US	05 2020	hand sanitizer, social distancing, COVID testing, contact tracing
Effenberger, Kronbichler, Shin, Mayer, Tilg, Perco (2020) [8]	Significant correlations were found between GT data relating to coronavirus and new COVID-19 cases across studied countries. The time lag was 11.5 days.	KR, JP, IR, IT, AT, DE, UK, US, EG, AU, BR, CN	12 2019–04 2020	Coronavirus (virus)
Lin, Liu, Chiu (2020) [61]	Google searches for “wash hands” from January to February correlated with lower COVID-19 spread from February to March in 21 countries.	IT, IR, KR, FR, ES, DE, US, CH, NL, SE, NO, AT, AU, CA, JP, UK, BE, SG, HK, TW, TH	01 2020–02 2020	wash hands, face mask
Brunori and Resce (2020) [15]	Significant positive correlation found between google search queries of COVID-19 symptoms and reported COVID-19 deaths.	IT	02 2020–03 2020	‘fever’, ‘dry cough’, ‘cough’, ‘sore throat’, ‘loss of sense of smell’, and ‘loss of sense of taste’
Sulyok, Ferenci, Walker (2021) [16]	Strong positive correlation found between Google search queries for coronavirus and COVID-19 cases in Europe.	BE, FE, DE, HU, IE, IT, NL, NO, ES, SE, CH, UK	01 2020–03 2020	Coronavirus
Abbas, Morland, Hall, El-Manzalawy (2021) [47]	The dynamics of the correlations found between GT data COVID-19 cases and deaths suggest that it would be possible to make predictions of COVID-19 cases and mortality rates up to 3 weeks in advance.	US	Dataset released 09 2020, accessed 11 2020	422 symptoms and conditions dataset. Appendix B.
Pellegrini, Ferrucci, Guaraldi, Bernabei, Scorcia, Giannaccare (2021) [17]	GT data on conjunctivitis reveals significant correlations with COVID-19 new cases with a lag of 14–18 days.	IT, FR, UK, US	01 2020–04 2020	“conjunctivitis” and the translation in Italian (“congiuntivite”) and French (“conjonctivite”)
Yousefinaghani, Dara, Mubareka, Sharif (2021) [48]	GT data allowed to identify starts and peaks of COVID-19 waves 1 and 3 weeks earlier, respectively. Strong correlation was found between Twitter/GT data and the number of COVID-19 cases in Canada with 3–5-week lags.	CA, US	01 2020–09 2020	Shortness of breath, cough, fever, sore throat, loss of smell, loss of taste, face mask, quarantine, wearing mask, wash hand, COVID-19 vaccine, COVID-19 vaccine, covid vaccine, corona vaccine, coronavirus vaccine, physical distancing, social distancing
Cinarka, Uysal, Cifter, Niksarlioglu, Çarkoğlu (2021) [18]	Online interest shown in COVID-19 pulmonary symptoms can reliably predict later reported cases of the first COVID-19 wave.	TR, IT, ES, FR, UK	01 2020–08 2020	fever, cough, dyspnea
Husnayain, Chuang, Fuad, Su (2021) [49]	Significant correlations between COVID-19 and GT data reached their highest point in June and decreased as the outbreak progressed.	US	01 2020–12 2020	Data retrieved for COVID-19-related terms, topics, and disease; the top related queries; most-searched COVID-19 terms in 2020 with a lag of 7 days
Kristensen, Lorenz, May, Strauss, (2021) [19]	Significant correlations found between term “RKI” and increase in COVID-19 cases (2–12-day lag). Similar pattern was observed for the term “corona”. Searches for “protective mask” peaked 6–12 days after the peak of COVID-19 cases.	DE	02 2020–04 2020	‘RKI’ (Robert Koch Institut), ‘Mundschutz’ (protective mask), and ‘corona’
Hu, Lou, Xu, Meng, Xie, Zhang, Zou, Liu, Sun, Wang (2020) [34]	Slightly positive significant correlation found between GT data regarding COVID-19 and daily confirmed COVID-19 cases.	US, UK, CA, IE, AU, NZ	12 2019–02 2020	2019-nCoV + SARS-CoV-2 + novel coronavirus + new coronavirus + COVID-19 + Corona Virus Disease 2019
Schuster, Tizek, Schielein, Ziehfreund, Rothe, Spinner, Biedermann, Zink (2021) [33]	Moderate correlation found between GT data and confirmed new COVID-19 cases over the study period.	DE	01 2020–07 2020	coronavirus
Li, Chen, Chen, Zhang, Pang, Chen (2020) [59]	Internet search terms had high correlation with daily COVID-19 cases.	CN	01 2020–02 2020	coronavirus, pneumonia
Walker, Sulyok (2020) [32]	Search terms related to coronavirus had a significant correlation with confirmed COVID-19 cases.	UK	01 2020–04 2020	Coronavirus (virus), hand washing (search term), and face mask (search term)
Samadbeik, Garavand, Aslani, Ebrahimzadeh, Fatehi (2022) [55]	Terms related to COVID, COVID-19, and coronavirus had a significant correlation with confirmed weekly COVID-19 cases.	IR	02 2020–01 2021	corona [Persian], Covid [Persian], COVID-19, corona, and coronavirus
Ahmed, Abid, de Oliveira, Ahmed, Siddiqui, Siddiqui, Jafri, Lippi (2021) [54]	‘Loss of smell’ was the best predictor for positive weekly COVID-19 cases.	PK	03 2021–06 2021	Fever, cough, headache, shortness of breath, taste loss, and hearing loss, COVID-19, coronavirus, virus, COVID
Yuan, Xu, Hussain, Wang, Gao, Zhang (2020) [51]	COVID-19 search terms had a strong correlation with confirmed COVID-19 cases and deaths in the USA.	US	03 2020–04 2020	COVID-19, COVID, coronavirus, SARS-CoV-2, pneumonia, high temperature, cough, COVID heart, COVID pneumonia, and COVID diabetes
Aragón-Ayala, Copa-Uscamayta, Herrera, Zela-Coila, Cender Udai Quispe-Juli (2021) [62]	Most countries showed a moderate to strong significant correlation between COVID-19 searches and daily new cases.	AR, BO, BR, CL, CO, CR, CU, EC, SV, GT, HN, MX NI, PA, PY, PE, PR, DO, UY, VE	12 2019–04 2020	“coronavirus + COVID-19 + SARS-CoV2 + nuevo coronavirus + 2019-nCoV”, “coronavirus + coronavírus + COVID-19 + SARS-CoV2 + novo coronavirus + novo coronavírus + 2019-nCoV”

TW—Taiwan, IT—Italy, ES—Spain, UK—United Kingdom, US—United States, DE—Germany, FR—France, NL—Netherlands, IR—Iran, IN—India, CN—China, BR—Brazil, AU—Australia, CA—Canada, ZA—South Africa, PL—Poland, PT—Portugal, KR—Republic of Korea, JP—Japan, AT—Austria, EG—Egypt, CH—Switzerland, SE—Sweden, NO—Norway, BE—Belgium, SG—Singapore, HK—Hong Kong, TH—Thailand, HU—Hungary, TR—Turkey, IE—Ireland, AR—Argentina, BO—Bolivia, CL—Chile, CO—Columbia, CR—Costa Rica, CU—Cuba, EC—Ecuador, SV—El Salvador, GT—Guatemala, HN—Honduras, MX—Mexico, NI—Nicaragua, PA—Panama, PY—Paraguay, PE—Peru, PR—Puerto Rico, DO—Dominican Republic, UY—Uruguay, VE—Venezuela, and PK—Pakistan.

**Table 2:** Publications where GT data were analyzed using more complex methods.

Author and Year	The Main Findings about Google Trends	Country	Period	Keywords
Ayyoubzadeh, Zahedi, Ahmadi, Niakan Kalhori (2020) [53]	Data mining algorithms (linear regression and long short-term memory) can predict COVID-19 outbreak trends.	IR	02 2020–03 2020	Corona, COVID-19, Coronavirus, Antiseptic selling, Antiseptic buying, Hand washing, Hand sanitizer, Ethanol, Antiseptic
Prasanth, Singh, Kumar, Tikkiwal, Chong (2021) [63]	Data obtained from GT significantly improved deep learning model (long short-term memory optimized with Grey Wolf optimization) for forecasting COVID-19 numbers.	IN, US, UK	02 2020–05 2020	Coronavirus symptoms, Coronavirus, Covid, Hand wash, Healthcenter, Mask, Positive cases, Sanitizer, Coronavirus vaccine
Niu, Liang, Zhang, Zhang, Qu, Su, Zheng, Chen et al. (2021) [21]	GT data combined with Adaboost algorithm had strong predictive ability of COVID-19 infection with hopes to further enhance the online prediction system.	IT	02 2020–03 2020	40 keywords. Appendix C.
Peng, Li, Rong, Chen, Chen (2020) [22]	A model with GT data and Random Forest Classification, developed from 20 countries worldwide, can be used for epidemic alert level prediction.	202 countries. Appendix D.	01 2020–04 2020	Coronavirus, Pneumonia, Cough, Diarrhea, Fatigue, Fever, Nasal congestion and Rhinorrhea
Rabiolo, Alladio, Morales, McNaught, Bandello, Afifi, Marchese et al. (2021) [23]	GT data could improve statistical models (ERS, ARIMA, and NNA models fitted on the first two principal components) of nowcasting and forecasting COVID-19 incidence with a 15-day time lag and could be used as one of surveillance systems for this disease.	AU, BR, FR, IN, IR, ZA, UK, US	01 2015–07 2020 (weekly data) and 01 2020–12 2020 (daily data)	20 topics: abdominal pain, ageusia, anorexia, anosmia, bone pain, chills, conjunctivitis, cough, diarrhea, eye pain, fatigue, fever, headache, myalgia, nasal congestion, nausea, rhinorrhea, shortness of breath, sore throat, and tearing
Turk, Tran, Rose, McWilliams (2021) [50]	GT data were incorporated in a vector error correction model, which showed very good results in forecasting regional COVID-19 hospital census.	US	02 2020–08 2020	Coronavirus, covid testing + covid test + covid19 Testing + covid19 test + covid 19 Testing + covid 19 test, headache, pneumonia, “shortness of breath” + “trouble breathing” + “difficulty breathing”, CDC
Peng, Li, Rong, Pang, Chen, Chen (2021) [25]	Random forest regression algorithm with integrated previous incidence and GT data was able to accurately predict increase in COVID-19 cases in most countries 7 days in advance.	215 countries. Appendix E.	01 2020–07 2020	Fourteen terms, including coronavirus, pneumonia, and COVID-19; six symptom-related terms (cough, diarrhea, fatigue, fever, nasal congestion, and rhinorrhea); five prevention-related terms (hand washing, hand sanitizer, mask, social distance, and social isolation)

IR—Iran, IN—India, US—United States, UK—United Kingdom, IT—Italy, AU—Australia, BR—Brazil, FR—France, and ZA—South Africa.

**Table 3:** Publications with negative results of GT use for COVID-19 prediction and surveillance.

Author and Year	The Main Findings about Google Trends	Country	Period	Keywords
Szmuda, Ali, Hetzger, Rosvall, Słoniewski (2020) [26]	GT data did not correlate with COVID-19 incidence and mortality; however, they had a strong correlation with international WHO announcements.	40 European countries. Appendix F.	12 2019–04 2020	Coronavirus
Asseo, Fierro, Slavutsky, Frasnelli, Niv (2020) [27]	The correlation between internet searches for symptoms and new COVID-19 cases varied significantly over time. High fluctuations show that relying only on GT data to monitor the spread of COVID-19 is not a viable strategy.	IT, US	03 2020–04 2020	taste loss, smell loss, sight loss (control), hearing loss (control), COVID symptoms (and the same in Italian)
Muselli, Cofini, Desideri, Necozione (2021) [28]	The volume of Google searches did not reflect the actual epidemiological situation. It has been seen that official communications and government activity has more impact on public interest in the disease.	IT	12 2019–03 2020	coronavirus, coronavirus symptoms (in Italian), coronavirus news (in Italian), and coronavirus Italy (in Italian)
Rovetta (2021) [29]	Big number of anomalies seen in multiple cities’ relative search volumes (RSVs) made these data unusable for statistical inference. Furthermore, correlations varied greatly depending on the day RSVs were collected.	IT	02 2020–12 2020 and 02 2020–05 2020	coronavirus + covid
Satpathy, Kumar, Prasad (2021) [57]	Correlations found between GT queries and COVID-19 cases maybe either because of media-coverage-induced curiosity or health-seeking curiosity.	IN	01 2020–05 2020	88 terms in Hindi and English. Appendix G.
Sato, Mano, Iwata, Toda (2021) [30]	Results suggest that search keywords, previously identified as candidates for COVID-19 prediction, might be unreliable.	JP, AU, CA, UK, IE, IN, SG, US, ZA	10 2017–10 2020	54 English keywords and the corresponding 60 Japanese keywords. Appendix H.
Dagher, Lamé, Hubiche, Ezzedine, Duong (2021) [31]	Google searches for chilblain were influenced by media coverage and government policies during the COVID-19 pandemic, showing that GT, as a monitoring tool for emerging infectious diseases, should be used with caution.	US, UK, FR, IT, ES, DE	01 2020–05 2020	(1) toe or chilblains and (2) coronavirus,
Madden, Feldman (2021) [52]	Search terms do not give any evidences suggesting earlier COVID-19 spread.	US	09 2015–03 2020	Can’t smell OR can’t taste or smell OR why can’t i smell or taste OR why can’t i taste or smell anything
Sousa-Pinto, Anto, Czarlewski, Anto, Fonseca, Bousquet (2020) [62]	COVID-19-related searches are more closely related to media coverage than to ongoing COVID-19 epidemic.	RA, AU, BE, BR, CA, CL, FR, DE, IT, PT, RU, ES, SE, CH, NL, UK, US	2015 04–2020 05	coronavirus, cough, anosmia, ageusia

IT—Italy, US—United States, IN—India, JP—Japan, AU—Australia, CA—Canada, UK—United Kingdom, IE—Ireland, SG—Singapore, ZA—South Africa, FR—France, ES—Spain, DE—Germany, RA—Argentina, BE—Belgium, BR—Brazil, CL—Chile, PT—Portugal, RU—Russia, SE—Sweden, CH—Switzerland, and NL—The Netherlands.

## Data Availability

Not applicable.

## Appendix A

The screening library of 463 search queries was obtained using the GT “Related Queries” function on an initial bank of 23 coronavirus-related terms. Seed Terms: ‘am i sick’, ‘cdc’, ‘corona virus near me’, ‘coronavirus doctor’, ‘coronavirus help’, ‘coronavirus hospital’, ‘coronavirus symptoms’, ‘coronavirus testing’, ‘coronavirus treatment’, ‘coronavirus vs. flu symptoms’, ‘cough’, ‘covid 19 symptoms’, ‘covid 19 testing’, ‘do i have coronavirus’, ‘doctor near me’, ‘insurance coronavirus’, ‘sick’, ‘sore throat’, ‘symptoms coronavirus 2020’, and ‘testing’.

## Appendix B

The authors used 422 time-series datasets extracted from the Google COVID-19 Search Trends Symptoms dataset, which was released by Google on 2 September 2020 and is available at: https://github.com/google-research/open-COVID-19-data/ (accessed on 7 May 2022). The study authors accessed the dataset 6 November 2020.

## Appendix C

Keywords used in the correlation analysis: COVID-19, Novel coronavirus, Coronavirus, SARS, COVID-19 data, COVID-19 Italy, SARS-CoV-2, COVID-19 symptoms, Influenza, Pneumonia, Fever, Cough, Sore throat, Chest distress, Difficulty breathing, Fatigue, Fell sick and vomit, Diarrhoea, Muscle ache, Mask, Disinfect, Isolation, Protective suit, Goggle, Thermometer, Disposable gloves, Medical supplies, COVID-19 vaccine, N95, Confirmed cases, New cases, Suspected case, Infection, Epidemic, Fatality rate, ISS, WHO, Incubation period, Hospital, and Nurse.

## Appendix D

202 countries: Argentina, Australia, Austria, Belgium, Brazil, Finland, France, Germany, India, Indonesia, Iran (Islamic Republic of), Ireland, Italy, Peru, Poland, Puerto Rico, South Africa, Spain, Switzerland, the United States of America, Aruba, Central African Republic, French Polynesia, Ghana, Venezuela (Bolivarian Republic of), Canada, Chad, Colombia, Costa Rica, Coted Ivoire, Greece, Guadeloupe, Iceland, Kuwait, Morocco, Netherlands, Panama, Republic of Moldova, Rwanda, the United Kingdom, the United States Virgin Islands, Uruguay, Uzbekistan, Albania, Anguilla, Antigua and Barbuda, Bahrain, Bolivia (Plurinational State of), Botswana, Bulgaria, Burundi, Cameroon, Chile, Croatia, Cuba, Cyprus, Dominican Republic, Eritrea, Eswatini, Falkland Islands (Malvinas), French Guiana, Gambia, Grenada, Honduras, Kazakhstan, Kenya, Kyrgyzstan, Lebanon, Luxembourg, Malta, Martinique, Mauritania, Montenegro, Nigeria, Norway, Oman, Portugal, Qatar, Reunion, Saint Kitts and Nevis, Saint Martin, Serbia, Sierra Leone, Slovakia, Slovenia, Ukraine, Algeria, Armenia, Azerbaijan, Barbados, Belarus, Burkina Faso, Cayman Islands, Curacao, Czechia, Denmark, Ecuador, Egypt, El Salvador, Equatorial Guinea, Estonia, Guatemala, Guinea, Guinea-Bissau, Hungary, Jordan, Kosovo, Latvia, Lithuania, Malawi, Mali, Mauritius, Mexico, New Caledonia, Niger, Paraguay, Saint Barthelemy, Saint Vincent and the Grenadines, Senegal, Sint Maarten, Sweden, Togo, Tunisia, Turkey, Saudi Arabia, Bahamas, Bangladesh, Benin, Bermuda, Bhutan, Djibouti, Gibraltar, Guernsey, Guyana, Haiti, Jersey, Liberia, Libya, Madagascar, Montserrat, Mozambique, North Macedonia, Philippines, Romania, Russian Federation, Seychelles, Somalia, South Sudan, Turks and Caicos Islands, Uganda, the United Republic of Tanzania, Afghanistan, Pakistan, Sudan, Bosnia and Herzegovina, Fiji, Georgia, Greenland, Guam, Isle of Man, Jamaica, Japan, Liechtenstein, Myanmar, Nepal, Papua New Guinea, San Marino, Singapore, Sri Lanka, Suriname, the United Arab Emirates, Zambia, Zimbabwe, Andorra, Belize, Ethiopia, Gabon, Malaysia, Mayotte, Mongolia, Nicaragua, Saint Lucia, Sao Tome and Principe, Timor-Leste, Yemen, Angola, British Virgin Islands, Dominica, New Zealand, Thailand, Trinidad and Tobago, Brunei Darussalam, Cambodia, Faroe Islands, Iraq, Israel, Maldives, Northern Mariana Islands (Commonwealth of the), Syrian Arab Republic, China, Vietnam, and Laos.

## Appendix E

A total of 215 countries and territories: Afghanistan, Albania, Algeria, Andorra, Anguilla, Argentina, Armenia, Australia, Austria, Azerbaijan, Bahrain, Bangladesh, Belarus, Belgium, Bolivia (Plurinational State of), Bonaire, Sint Eustatius and Saba, Bosnia and Herzegovina, Brazil, British Virgin Islands, Brunei Darussalam, Bulgaria, Burkina Faso, Cabo Verde, Canada, Chad, Chile, China, Colombia, Congo, Costa Rica, Coted Ivoire, Croatia, Cuba, Cyprus, Czechia, Democratic Republic of the Congo, Denmark, Djibouti, Dominica, Dominican Republic, Ecuador, Egypt, El Salvador, Estonia, Eswatini, Ethiopia, Falkland Islands (Malvinas), Fiji, Finland, France, French Guiana, Georgia, Germany, Ghana, Greece, Greenland, Grenada, Guam, Guatemala, Guinea, Haiti, Honduras, Hungary, Iceland, India, Indonesia, Iran (Islamic Republic of), Iraq, Ireland, Italy, Jamaica, Japan, Jordan, Kazakhstan, Kenya, Kosovo, Kuwait, Laos, Latvia, Liberia, Lithuania, Luxembourg, Madagascar, Malawi, Malaysia, Mali, Mauritania, Mayotte, Mexico, Montenegro, Montserrat, Morocco, Mozambique, Myanmar, Namibia, Nepal, Netherlands, New Zealand, Niger, Nigeria, North Macedonia, Northern Mariana Islands (Commonwealth of the), Norway, occupied Palestinian territory, Oman, Pakistan, Panama, Paraguay, Peru, Philippines, Poland, Portugal, Puerto Rico, Qatar, Republic of Korea, Republic of Moldova, Reunion, Romania, Russian Federation, Saint Kitts and Nevis, Saint Lucia, Saint Pierre and Miquelon, San Marino, Saudi Arabia, Senegal, Serbia, Sierra Leone, Singapore, Slovakia, Slovenia, Somalia, South Africa, Spain, Sri Lanka, Suriname, Sweden, Switzerland, Tajikistan, the United Kingdom, Togo, Tunisia, Turkey, Uganda, Ukraine, the United Arab Emirates, the United States of America, Uruguay, Uzbekistan, Venezuela (Bolivarian Republic of), Yemen, Antigua and Barbuda, Bahamas, Bermuda, Burundi, Cambodia, Cameroon, Cayman Islands, Central African Republic, Comoros, Equatorial Guinea, Eritrea, Gabon, Gibraltar, Guernsey, Guinea-Bissau, Isle of Man, Israel, Jersey, Lebanon, Lesotho, Liechtenstein, Maldives, Mauritius, Monaco, Mongolia, New Caledonia, Nicaragua, Saint Vincent and the Grenadines, Sao Tome and Principe, Seychelles, South Sudan, Sudan, Syrian Arab Republic, Thailand, Timor-Leste, the United Republic of Tanzania, the United States Virgin Islands, Zambia, Angola, Barbados, Benin, Bhutan, Curacao, Libya, Malta, Rwanda, Trinidad and Tobago, Viet Nam, Zimbabwe, Belize, Botswana, Gambia, Guadeloupe, Martinique, Papua New Guinea, Saint Barthelemy, Faroe Islands, French Polynesia, Guyana, Kyrgyzstan, Sint Maarten, Turks and Caicos Islands, Aruba, Holy See, and Saint Martin.

## Appendix F

A total of 40 countries: Albania, Andorra, Austria, Belarus, Belgium, Bosnia and Herzegovina, Bulgaria, Croatia, Czechia, Denmark, Estonia, Finland, France, Germany, Greece, Holy See, Hungary, Iceland, Ireland, Italy, Latvia, Liechtenstein, Lithuania, Luxembourg, Malta, Moldova, Monaco, Montenegro, Netherlands, North Macedonia, Norway, Poland, Portugal, Romania, Russia, San Marino, Serbia, Slovakia, Slovenia, Spain, Sweden, Switzerland, Ukraine, and the United Kingdom. Holy See, Liechtenstein, Monaco, and San Marino were excluded due to being small and therefore having insufficient data from Google Trends.

## Appendix G

A total of 88 search terms: Coronavirus, Corona, Covid 19, Covid, SARS-CoV-2, SARS Novel coronavirus, Novel corona, Virus, Infection, Disease, Social distancing, Hand wash, Hand rub, Mask, Facemask, Sanitizer, Soap, Fever, Cough, Cold, Breathlessness, Fatigue, Rhinorrhoea, Nasal congestion, Sneeze, Myalgia, Sore throat, Diarrhoea, Anorexia, Chest pain, Headache, Nausea, Ageusia, Abdominal pain, Dizziness, Vomiting, Eye pain, Anosmia, Doctor, Nurse, Hospital, Clinic, Medicine, Check-up, OPD, Treatment, Testing, Lockdown, Quarantine, Isolation, Bhilwada model, Curfew, Diya, Thali, Warrior, Shop, Market, Open, Ticket, Rail, Bus, Modi, PM cares, 20 lakh, Kerala, Mumbai, *Khansi* (Cough), *Bukhar* (Fever), Dawa (Medicine), *Dawai* (Medicine), *Kharash* (Sore throat), *Sans* (Breathlessness), *Sardi* (Cold), *Jukam* (Cold), *Dama* (Asthma), coronavirus, Corona, Covid 19, Covid, Crona, Lockdown, Social distancing, *hath dhona* (Hand wash), *Mukhauta* (Mask), *Sabun* (Soap), *Deepak* (Lamp), and *Thali* (Plate).

## Appendix H

A total of 54 English keywords: Malaise, fatigue, tired, Anorexia, diarrhea, constipation, Abdominal pain, stomach ache, nausea, Chest pain, dyspnea, vomiting, Shortness of breath, short of breath, pneumonia, Cough, sputum, rhinitis, Runny nose, nasal discharge, study nose, Sneeze, sore throat, throat pain, Fever, chills, cold, Sense of smell, loss of smell, anosmia, Sense of taste, loss of taste, dysgeusia, Hair loss, loss of hair, bald, Myalgia, muscle pain, body aches, Arthralgia, joint pain, pain, Eye pain, sore, congestion, Headache, memory loss, confusion, Vertigo, dizziness, dizzy, Insomnia, anxiety, and numbness. Corresponding Japanese keywords can be found at: https://www.ncbi.nlm.nih.gov/pmc/articles/PMC8286439/bin/12874_2021_1338_MOESM1_ESM.docx (accessed on 7 May 2022).
